# Longitudinal clinical study of patients with iron rim lesions in multiple sclerosis

**DOI:** 10.1177/13524585221114750

**Published:** 2022-08-24

**Authors:** Amjad I Altokhis, Aimee M Hibbert, Christopher M Allen, Olivier Mougin, Abdulmajeed Alotaibi, Su-Yin Lim, Cris S Constantinescu, Rasha Abdel-Fahim, Nikos Evangelou

**Affiliations:** Mental Health and Clinical Neuroscience Academic Unit, University of Nottingham, Nottingham, UK/Department of Neurology, Nottingham University Hospital NHS Trust, Nottingham, UK/Division of Health and Rehabilitation Sciences, Princess Nourah Bint Abdulrahman University, Riyadh, SA; Department of Neurology, Nottingham University Hospital NHS Trust, Nottingham, UK; Mental Health and Clinical Neuroscience Academic Unit, University of Nottingham, Nottingham, UK/Department of Neurology, Nottingham University Hospital NHS Trust, Nottingham, UK; Sir Peter Mansfield Imaging Centre, School of Medicine, University of Nottingham, Nottingham, UK; Mental Health and Clinical Neuroscience Academic Unit, University of Nottingham, Nottingham, UK/Department of Neurology, Nottingham University Hospital NHS Trust, Nottingham, UK/Department of Radiological Sciences, School of Applied Medical Sciences, King Saud bin Abdul-Aziz University for Health Sciences, Riyadh, Saudi Arabia/Radiology and Neurosciences unit, King Abdullah International Medical Research Centre, Riyadh, Saudi Arabia; Mental Health and Clinical Neuroscience Academic Unit, University of Nottingham, Nottingham, UK/School of Medicine, Faculty of Health and Medical Sciences, Taylor’s University, Subang Jaya, Malaysia; Department of Neurology, Cooper Neurological Institute, Camden, NJ, USA; Department of Neurology, Nottingham University Hospital NHS Trust, Nottingham, UK; Mental Health and Clinical Neuroscience Academic Unit, University of Nottingham, Nottingham, UK/Department of Neurology, Nottingham University Hospital NHS Trust, Nottingham, UK

**Keywords:** Multiple sclerosis (MS), magnetic resonance imaging (MRI), white matter lesion (WML), iron rim lesion (IRL), biomarkers

## Abstract

**Background:**

Iron rims (IRs) surrounding white matter lesions (WMLs) are suggested to predict a more severe disease course. Only small longitudinal cohorts of patients with and without iron rim lesions (IRLs) have been reported so far.

**Objective:**

To assess whether the presence and number of IRLs in patients with clinically isolated syndrome (CIS) and multiple sclerosis (MS) are associated with long-term disability or progressive disease.

**Methods:**

Ninety-one CIS/MS patients were recruited between 2008 and 2013 and scanned with 7 T magnetic resonance imaging (MRI). Expanded Disability Status Scale (EDSS) was used to calculate Age-related Multiple Sclerosis Severity Score (ARMSS) at the time of scan and at the latest clinical follow-up after 9 years. WMLs were assessed for the presence of IRL using Susceptibility weighted imaging (SWI)-filtered phase images.

**Results:**

In all, 132 IRLs were detected in 42 patients (46%); 9% of WMLs had IRs; 54% of the cohort had no rims, 30% had 1–3 rims and 16% had ⩾4. Patients with IRL had a higher EDSS and ARMSS. Presence of IRL was also a predictor of long-term disability, especially in patients with ⩾4 IRLs. IRLs have a greater impact on disability compared to the WML number and volume.

**Conclusion:**

The presence and number of perilesional IR on MRI hold prognostic value for long-term clinical disability in MS.

## Introduction

Multiple sclerosis (MS) is one of the leading causes of disability in young adults.^1^ Given the availability of multiple disease-modifying therapies (DMTs) and different approaches to MS treatment, there is a need to predict long-term disability.^2^ Neurologists frequently select DMT based on the presence of prognostic factors.^3^ Yet, there are still few prognostic markers that can be easily used in a routine clinical setting.^4^

Iron accumulation at the edge of white matter lesions (WMLs) represent an emerging imaging biomarker that reflects iron-laden microglia and macrophages present in perilesional chronic inflammation.^5–9^ Iron rim lesions (IRLs) are associated with remyelination failure and subsequent axonal loss. They appear as hypointense, ring-like structures on 7 T or 3 T magnetic resonance imaging (MRI) susceptibility imaging sequences.^10,11^

MRI^7^ and neuropathological^10,12^ studies have reported that IRLs are present in more than half of MS patients and have been associated with clinical disability. It has been speculated that IRLs could serve as an imaging biomarker to predict future physical disability in MS.^13,14^ Two recent studies reported that patients with IRLs had more aggressive disease course but their conclusions are tempered by either their sample size or follow-up duration.^13,15^

In this large retrospective study, we aim to evaluate the association of IRLs on 7 T SWI-filtered phase images^9^ with clinical outcomes after a long clinical follow-up. Specifically, we aim to explore (1) whether the presence and number of IRL on an MRI scan is a predictor of worse clinical disability, (2) IRL presence in different MS disease phenotypes and (3) the association between IRL number and WML count and volume.

## Materials and methods

### Clinical cohort

Between August 2008 and July 2013, 156 patients with neuroinflammatory conditions were recruited from the outpatient neurology clinic at the Queen’s Medical Centre in Nottingham to participate in ultra-high field MRI research studies.

**Figure 1.**
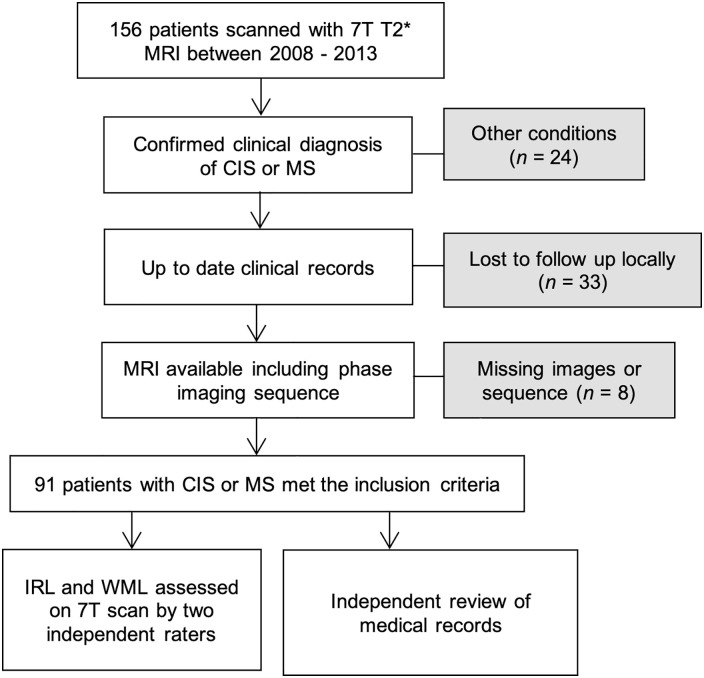
Flowchart summarises the study selection.

Inclusion criteria used in this longitudinal clinical follow-up were aged 18 years or older, diagnosis of clinically isolated syndrome (CIS) or MS according to the revised McDonald criteria,^16,17^ up-to-date clinical records and the availability of 7 T MRI scan including SWI (Figure 1). CIS and MS patients were included independently of their MRI findings. Patient demographics and clinical characteristics were collected by the research MS team (experienced MS neurologists and their MS fellows) at the time of their 7 T MRI scan: age, sex, disease duration, treatment duration, disease subtype and Expanded Disability Status Scale (EDSS).^18^ The Age-related Multiple Sclerosis Severity Score (ARMSS)^19^ was calculated. The latest clinical follow-up data were collected from hospital records in July 2021 to assess disability change. All studies contributing data to this longitudinal clinical follow-up received local ethics committee approvals, and all participants consented to have their clinical records reviewed.

### MRI acquisition

MRI scans were performed on a 7 T MRI scanner (Philips Medical Systems, The Netherlands). We used T1-TFE (IR-TFE) sequence (also known as MPRAGE) and 3D-FFE (3D-FLASH). High-resolution MPRAGE images were acquired with a tailored inversion pulse to reduce the effects of B1 inhomogeneity^20^ (TI = 1070 ms, FA = 8°, TE/TR = 7/15 ms for a total of 280 slices at a resolution 60 of 0.5 ′ 0.5 ′ 0.5 mm^3^ isotropic, for a total FOV of 205 ′ 215 ′ 140 mm and acquisition time of 11 minutes). 3D T2* weighted EPI images were acquired with a 3D FLASH-T2* weighted spoiled Gradient Echo sequence (TR/TE = 150/20 ms, flip angle = 14°, FOV of 216 × 216 × 85 mm, with a resolution of 0.5 × 0.5 × 0.5 mm^3^ and acquisition time of 9 minutes). Both magnitude and phase were saved, and the SWI-filtered phase images were reconstructed off-line using a high-pass filter as described in this study.^9^

### Lesion identification

**Figure 2.**
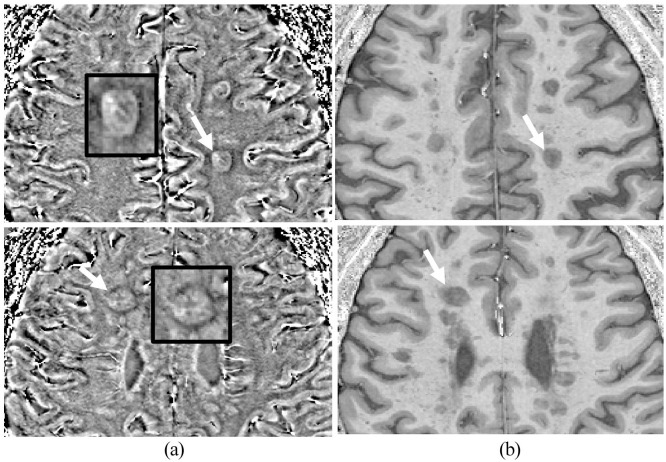
(a) Typical appearance of two lesions with rims on SWI-filtered phase image corresponding with (b) T1-weighted (MPRAGE). Enlarged images of the lesions indicated using the white arrows from different patients are shown in the black box with the characteristic hypointense rim surrounding most of the lesion.

For each patient, the number and volume of WML^21,22^ were detected on T1 (MPRAGE) sequence. Previous work identified the superior sensitivity of 7 T MPRAGE in detecting MS WML compared to 3 T FLAIR MRI.^23^ The total lesion volume was recorded in mm^3^. After two-stage training by a neuroradiologist with MS experience, A.I.A. used the SWI-filtered phase images for rim detection. Inter-rater reproducibility was calculated on 10 random scans. A rim positive lesion (IRL+) was defined as a hypointense rim that surrounds more than 50% of the lesion margin and visible on at least three slices^24,25^ (Figure 2). Total IRLs number and volume were calculated for each participant. ITK-SNAP^26^ was used for image analysis, detecting lesions and manually calculating the volumes. First, T1 (MPRAGE) images were reviewed to detect and calculate the total volume of WML. Then, each lesion was individually checked for IRL presence on SWI images. The presence of rims was checked twice, by displaying two windows of SWI and T1 images side by side and by co-registering/overlapping the two images, to check for co-registration errors. Manual segmentation of the IRL was then performed to calculate the volume using the polygon feature. The IRLs were manually segmented in all the slices that the rim was visible. The volume of each rim lesion was then calculated.

### Statistical analysis

Statistical analysis was performed using Jamovi (Version 1.6) and SPSS software package (version 27.0). Reported summary statistics include means, odds ratios and relative risks along with associated 95% confidence intervals (CIs). Shapiro tests and visual inspection of histograms were used to assess normality of the variables. The association between IRLs presence and disability was initially assessed using Spearman’s rank correlation coefficient. Disease subtypes and IRLs presence and number were assessed with paired-sample *t*-test. The association between the presence and number of IRLs and disability (EDSS/ARMSS) was assessed using linear regression. In line with a previous IRLs study,^13^ we classified all patients into three groups based on the number of IRLs (0, 1–3 and ⩾4) to test their association with disability using linear regression. Receiver operating characteristic (ROC) analysis was used to assess, for a given IRLs threshold, the sensitivity and specificity to classify two patient groups: either an increase in or a decrease or unchanged ARMSS at follow-up. We calculated the change in ARMSS from baseline to follow-up and dichotomised patients into two groups; those with positive change (ARMSS at follow-up was greater than baseline) and those with negative or stable change (ARMSS at follow-up was either lower or the same as baseline).

Unpaired test variables were assessed using Wilcoxon test, since most of the variables were non-normally distributed. Mediation analysis is a method used to explain the process by which one variable affects another.^27^ A bootstrapping of 5000 samples was performed to explore whether IRLs number or WML number/volume has a greater direct effect on long-term disability. Statistical significance was set at *p* < 0.05. Intraclass correlation coefficient (ICC) was used to assess reliability for IRLs detections in 10 randomly selected scans.

## Results

### Clinical cohort and IRLs

**Figure 3.**
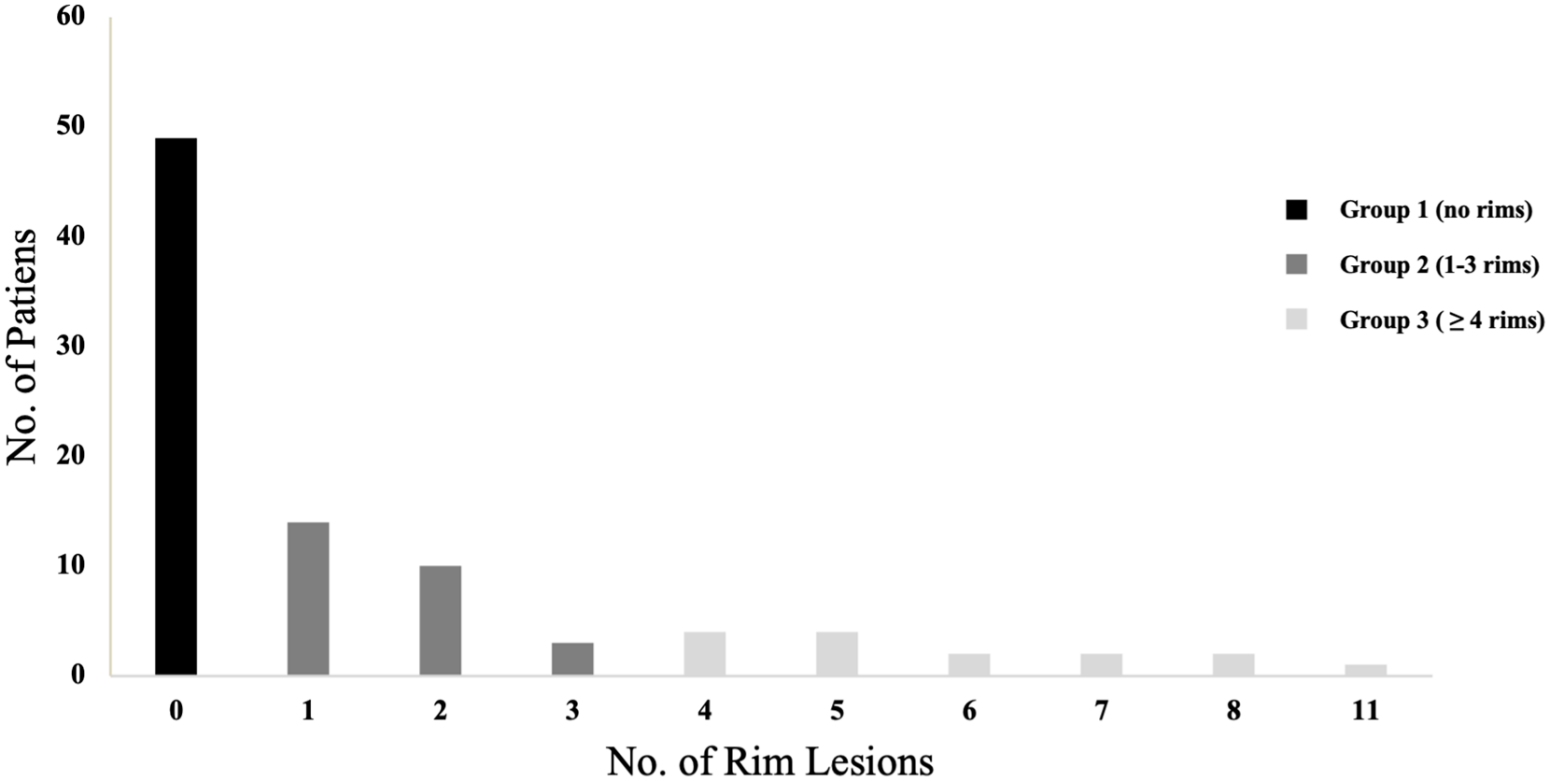
Histogram illustrating the number of iron rim lesions. Lesions were classified into three groups based on rim presence.

Of 156 patients with neuroinflammatory conditions scanned on 7 T MRI, data from 91 CIS and MS patients met the study inclusion criteria. Patients had a median of 7 years (IQR 2–15) from disease onset to the MRI scan; the median clinical follow-up after the MRI scan was 9 years (IQR 7–10). Nine percent of the 1468 WMLs had IR. Eighteen CIS patients had MS diagnosis at follow-up. Sex and age did not appear to affect the presence of IR. Study cohort demographic and clinical characteristics are presented in Table 1. Forty-nine patients (53.8%) had no rims (IRL–), 27 (29.6%) had one to three rims and 15 (16.4%) had four or more rims (Table 2, Figure 3). IRL+ patients had a median number of two IRLs (IQR 1–4). The intra- and inter-rater agreement was ICC = 0.95 and 0.81, respectively.

**Table 1.** Baseline demographic, clinical and imaging data of the study cohort.

	Total cohort	CIS	RRMS	SPMS	PPMS	IRL+	IRL–
Clinical and demographic data							
Patients, *n*	91	22	34	17	18	42	49
Female sex, *n* (%)	53 (58%)	13 (59%)	25 (74%)	9 (53%)	9 (50%)	27 (51%)	29 (55%)
Age, y	46 (18–75)	39 (18–64)	41 (20–58)	50 (33–61)	51 (32–75)	48 (23–75)	40 (18–65)
Disease duration, y	7 (0–40)	1 (0–22)	9 (0–32)	17 (4–40)	6 (1–17)	8 (0–40)	5 (0–32)
EDSS	4 (0–7)	1 (0–7)	2.5 (0–6.5)	6 (4.5–7.5)	5.5 (2.5–6.5)	4 (0–7)	3 (0–7)
ARMSS	5.4 (0.2–9.7)	1.8 (0.4–8.3)	4.9 (0.2–9.6)	7.4 (5.3–9.7)	6.25 (3.1–9.0)	6.7 (0.3–9.3)	5.0 (0.2–9.7)
DMT exposure, *n* (%)	27 (30%)	0	16 (47%)	11 (65%)	0	16 (38%)	11 (22%)
Duration on DMT before baseline, m	0 (0–140)	0	0.5 (0–130)	12 (0–140)	0	0 (0–130)	0 (0–140)
MRI data							
IRL+, *n*	42	11	13	12	6	42	NA
IRL count, *n* (F/M)	132 (88/44)	25 (16/9)	41 (30/11)	58 (37/21)	8 (5/3)	132 (88/44)	NA
WML count, *n* (% IRL)	1468 (9%)	171 (15%)	463 (9%)	526 (11%)	308 (3%)	1076 (12%)	392 (NA)
WML volume mm^3^, *n* (% with IRL)	135,179 (12%)	19,296 (20%)	38,019 (14%)	50,828 (9%)	27,036 (2%)	97,610 (19%)	37,279 (NA)
Total IRL volume mm^3^, *n*	15,509	4003	5412	4732	596	18,582	NA

All data presented as medians and ranges; IRL+: iron rim lesion positive; IRL–: iron rim lesion negative; EDSS: Expanded Disability Status Scale; ARMSS: Age-related Multiple Sclerosis Severity Score; DMT: disease-modifying treatment; CIS: clinically isolated syndrome; RRMS: relapsing remitting multiple sclerosis; SPMS: secondary progressive multiple sclerosis; PPMS: primary progressive multiple sclerosis. DMT patients treated refer to individuals who ever received treatment prior to scan. NA: not applicable.

**Table 2.** Cohort characteristics and their lesion analysis at baseline scan.

Characteristic	IRL–	1–3 IRL+	⩾4 IRL+
No. (%)	49 (53.8%)	27 (29.6%)	15 (16.4%)
Sex, F, *n* (%)	29 (58%)	18 (66.7%)	10 (66.7%)
Age, y	40 (18–65)	40 (21–75)	48 (23–64)
EDSS	3 (0–7)	2.5 (0–6.5)	6 (0–7)
ARMSS	6.5 (0.30–9.30)	4.9 (0.7–8.86)	8.1 (0.30–9.30)
Disease duration, y	5 (0–32)	6 (0–28)	9 (2–40)
Total rim lesion volume mm^3^, *n*	NA	6550	8115
Total lesion volume mm^3^, *n*	37,279	44,307	53,593

IRL: iron rim lesion positive; EDSS: Expanded Disability Status Scale; ARMSS: Age-related Multiple Sclerosis Severity Score; NA: not applicable.

### Association between IRLs and disease phenotype/evolution

**Figure 4.**
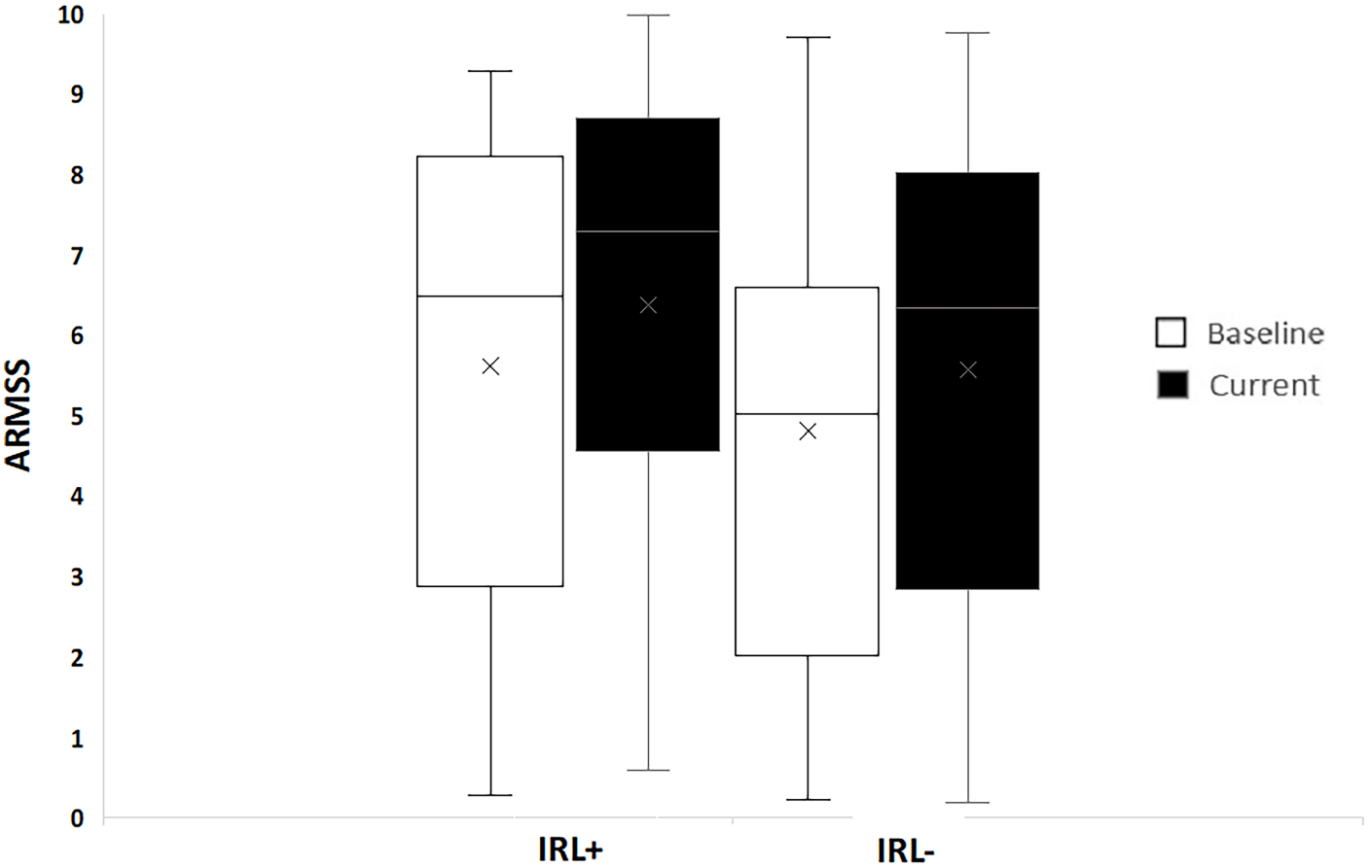
ARMSS at baseline and current clinical follow-up in patients with and without IRL. The median baseline ARMSS of the whole cohort was 5.4 (IQR 2.8–7.6). Patients with at least one IRL had a higher baseline ARMSS (6.7; IQR 3.1–8.1) compared to those without IRL (5.0; IQR 2.2–6.5).

The presence of IRLs was affected by disease subtype. A higher proportion of patients with secondary progressive multiple sclerosis (SPMS) had at least 1 IRL (Table 1), SPMS patients also had more rims per patient compared to all the other disease subtypes (*p* = 0.007). Development of relapsing remitting multiple sclerosis (RRMS)/SPMS was not affected by the presence of IRLs in those with an initial diagnosis of CIS (9/11 vs 9/11; a risk ratio of 1). IRL+ RRMS patients had a higher rate of progression to SPMS during the follow-up period compared to IRL– (7/13 vs 7/21), giving a risk ratio of 1.6 (95% CI, 0.74–3.55). Both at baseline and follow-up, the ARMSS (and EDSS) were higher in IRL+ patients (Figure 4).

### Association between clinical disability and the number of IRLs

The number of IRLs correlated with the baseline ARMSS (*p* < 0.006) even when accounting for total WML volume (*p* < 0.04). Similarly, it correlated with current ARMSS (*p* < 0.003). However, due to the high correlation between IRLs and WML number (*r* = 0.58) and volume (*r* = 0.48), they were not modelled in the same regression analysis. Therefore, mediation analysis was performed and showed that the direct effect of IRLs number on current ARMSS was greater (63%) than lesion count (0.23 (bootstrap CI, 0.007–0.46)) and even greater (77%) than lesion volume (0.30 (bootstrap CI, 0.10–0.09)).

**Figure 5.**
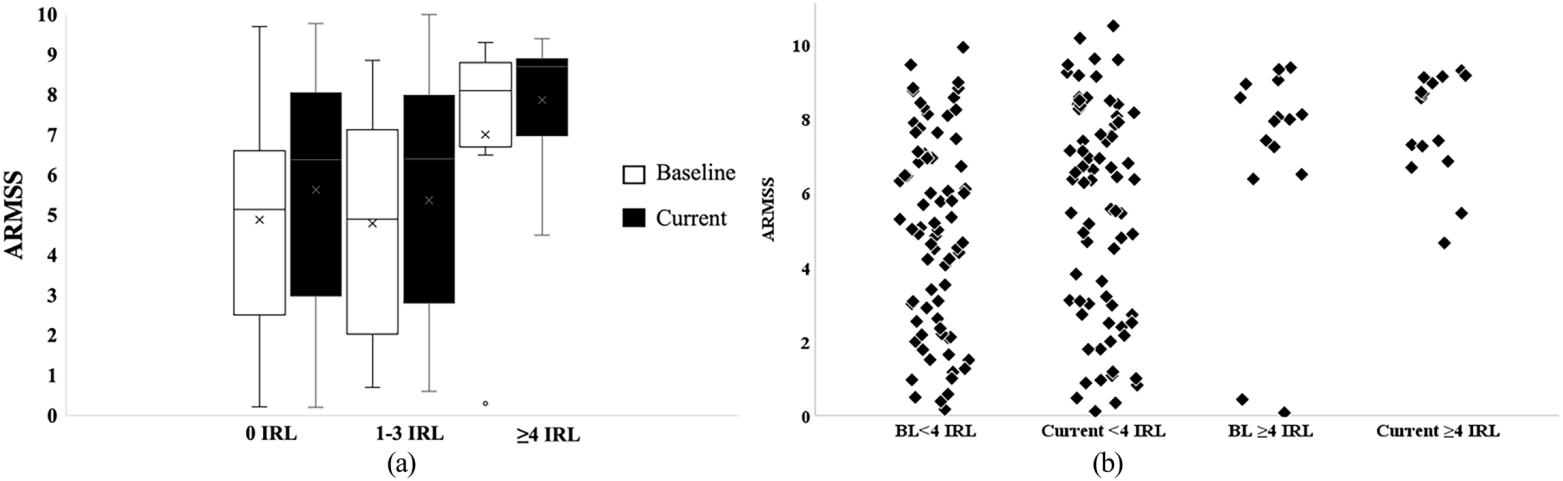
ARMSS at baseline and current clinical follow-up in IRL patients. The number of IRLs were grouped to (a; 0 IRLs, 1–3 IRLs and ⩾4 IRLs), (b; less than 4 IRLs and 4 or more rims).

**Figure 6.**
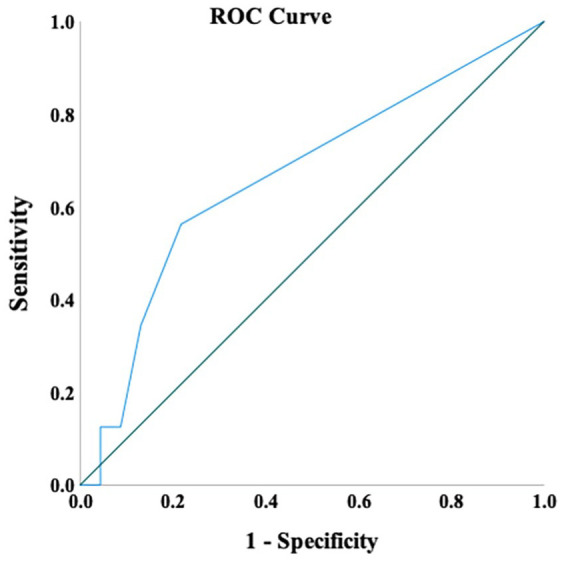
ROC curve for the cut-offs of iron rim lesions number. Reference line (in green).

Patients with ⩾4 IRLs had higher ARMSS at baseline (5.9) and follow-up (8.1) compared to those with <4 IRLs with ARMSS at baseline (5.4) and (6.7) at follow-up (Figure 5). Therefore, to explore whether it was a better prediction of disease progression in patients with ⩾4 IRLs, ROC analysis showed that a threshold of ⩾4 IRLs has a high specificity (95%) but showed low sensitivity (12%) to the rate of progression. The area under the curve (AUC) was 0.66 (*p* < 0.05) demonstrating poor prognostic accuracy (Figure 6). Thresholds lower than 4 IRL (not reported here) were less useful.

### IRLs and DMTs

IRLs were present despite prior treatment with DMT (copaxone, beta interferon and natalizumab). The odds of being IRL+/IRL– among previously treated patients was 16/26 and 12/37 in untreated patients, giving an odds ratio of 1.9 (95% CI, 0.77–4.67); the mean DMT duration prior to scanning was 16.8 months (SD = 32.8). Only two patients were treated with monoclonal antibody therapy (both natalizumab) prior to the scanning (both IRL+). The mean DMT duration prior to scanning and clinical follow-up was 22.2 (SD 35.4) months; RRMS was 54.7 (SD = 51.2) and SPMS 21.3 (SD = 36.6) months.

## Discussion

The role chronic active inflammation, reflected in the presence of IRLs in this study, plays in determining disability in MS patients has been a topic of interest in recent years.

In this retrospective study, we explored the association between the presence and number of IRLs on 7 T MRI with clinical outcomes, over a follow-up period of up to 12 years. We found that the presence of IRLs was associated with worsened clinical disability in a cohort of 91 patients, particularly if patients had more than four IRLs at their MRI scan, supporting the notion of unfavourable prognosis associated with IRLs presence.^13–15^

In line with others, at least one IRL was detected in almost half of our patients,^7,28^ and IRLs were more prevalent in SPMS patients compared to CIS and all other MS subtypes.^7,10,15,29–32^

We found patients with IRLs had a more aggressive disease (greater WML number and volume, higher disability scores at baseline and follow-up), corroborating findings from earlier studies.^12,13,15^ The contribution of our longer clinical follow-up study of 9 years validates the previously reported association of IRLs presence and long-term disability. We report patients with at least one rim lesion had higher disability^18^ and MS severity (accounting for patient age (global ARMSS)),^16^ comparable to two recent studies, Blindenbacher et al.^31^ followed-up 66 CIS and MS patients for a median of 2.9 years and Dal-Bianco et al.^15^ who follwed-up 8 MS patients for 7 years.

Although as a group, patients with IRLS do worse, we have found that the presence of one IRL does not appear to be sensitive in detecting patients with the worse outcome. In line with a recent report,^15^ we found that patients with ⩾4 IRLs had the worst disability scores, with high specificity.

As on average 9% of WM lesions have IR, we tested if identifying IRLs had an advantage over assessing the total WML load (count or volumes) in determining clinical disability. Previous studies did not explicitly account for this in their analysis. We performed mediation analysis and found that the number of IRLs had the most direct effect on disability compared to WML count and volume, supporting its role as an independent prognostic imaging biomarker.

Detecting and counting IRLs are much easier than assessing all WML in clinical settings. We have found IRLs present in DMT treated and untreated patients. We have to treat this observation very cautiously as the duration of DMT treatment was only 17 months and most patients were treated with first line agents. Although IRLs number was higher in patients ever treated with DMTs, this might also be reflected by the number/volume of WMLs, as patients with more extensive diseases are more likely to be treated. It remains to be seen whether long-term use of DMTs or higher effectiveness DMTs influence IRLs and more importantly whether long-term clinical disability can be mediated through IRLs.

We have found that iron-sensitive MRI may become an additional tool for monitoring and predicting disease progression, above what can be achieved through the study of gadolinium-enhanced lesions and brain atrophy alone.^15^

### Limitations and future direction

This study has limitations that include the exclusion of a large subset of patients due to the lack of clinical follow-up data. Also due to the retrospective study design, we were not able to accurately record all previous DMT data. Detecting IRLs on brain MRI is subjective and requires clear guidelines on manual assessment or automatic algorithms before these findings can be translated to clinical practice.

Our results support the need for (1) prospective studies using IRLs as a prognostic imaging biomarker in MS, (2) longitudinal studies to capture the long-term IRLs evolution, (3) study of the association between IRLs presence and disease progression in the spinal cord, (4) clinical trials to assess to what extent IRLs can practically contribute to monitoring disease progression and (5) validation of our results using 3 T clinical scanners as 7 T MRI is still limited to tertiary MS research centres.

## Conclusion

The presence and number of IRLs at MRI scan, especially the presence of ⩾4 rims, hold prognostic value for long-term clinical disability in MS. The effect of IRLs on disability was greater than WML number or volume. This supports the role of IRLs as an imaging biomarker for disease severity, which could be easily implemented in clinical practice.

## References

[1] Rosati G . The prevalence of multiple sclerosis in the world: An update. Neurol Sci 2001; 22(2): 117–139. 11603614 10.1007/s100720170011

[2] Ontaneda D Tallantyre E Kalincik T , et al. Early highly effective versus escalation treatment approaches in relapsing multiple sclerosis. Lancet Neurol 2019; 18(10): 973–980. 31375366 10.1016/S1474-4422(19)30151-6

[3] Rotstein D Montalban X . Reaching an evidence-based prognosis for personalized treatment of multiple sclerosis. Nat Rev Neurol 2019; 15(5): 287–300. 30940920 10.1038/s41582-019-0170-8

[4] Allen CM Mowry E Tintore M , et al. Prognostication and contemporary management of clinically isolated syndrome. J Neurol Neurosurg Psychiatry 2021; 92(4): 391–397. 10.1136/jnnp-2020-323087 33361410

[5] Absinta M Sati P Schindler M , et al. Persistent 7-tesla phase rim predicts poor outcome in new multiple sclerosis patient lesions. J Clin Invest 2016; 126(7): 2597–2609. 27270171 10.1172/JCI86198 PMC4922708

[6] Dal-Bianco A Grabner G Kronnerwetter C , et al. Slow expansion of multiple sclerosis iron rim lesions: Pathology and 7T magnetic resonance imaging. Acta Neuropathol 2017; 133(1): 25–42. 27796537 10.1007/s00401-016-1636-z PMC5209400

[7] Maggi P Sati P Nair G , et al. Paramagnetic rim lesions are specific to multiple sclerosis: An international multicenter 3T MRI study. Ann Neurol 2020; 88(5): 1034–1042. 32799417 10.1002/ana.25877 PMC9943711

[8] Absinta M Maric D Gharagozloo M , et al. A lymphocyte-microglia-astrocyte axis in chronic active multiple sclerosis. Nature 2021; 597(7878): 709–714. 34497421 10.1038/s41586-021-03892-7 PMC8719282

[9] Haacke EM Makki M Ge Y , et al. Characterizing iron deposition in multiple sclerosis lesions using susceptibility weighted imaging. J Magn Reson Imaging 2009; 29(3): 537–544. 19243035 10.1002/jmri.21676 PMC2650739

[10] Luchetti S Fransen NL van Eden CG , et al. Progressive multiple sclerosis patients show substantial lesion activity that correlates with clinical disease severity and sex: A retrospective autopsy cohort analysis. Acta Neuropathol 2018; 135(4): 511–528. 29441412 10.1007/s00401-018-1818-y PMC5978927

[11] Altokhis AI Alotaibi AM Felmban GA , et al. Iron rims as an imaging biomarker in MS: A systematic mapping review. Diagnostics 2020; 10(11): 968. 10.3390/diagnostics10110968 PMC7698946 33218056

[12] Frischer JM Weigand SD Guo Y , et al. Clinical and pathological insights into the dynamic nature of the white matter multiple sclerosis plaque. Ann Neurol 2015; 78(5): 710–721. 26239536 10.1002/ana.24497 PMC4623970

[13] Absinta M Sati P Masuzzo F , et al. Association of chronic active multiple sclerosis lesions with disability in vivo. JAMA Neurol 2019; 76(12): 1474–1483. 31403674 10.1001/jamaneurol.2019.2399 PMC6692692

[14] Marcille M Hurtado Rúa S Tyshkov C , et al. Disease correlates of rim lesions on quantitative susceptibility mapping in multiple sclerosis. Sci Reports 2022; 12(1): 1–10, https://www.nature.com/articles/s41598-022-08477-6 10.1038/s41598-022-08477-6 PMC8924224 35292734

[15] Dal-Bianco A Grabner G Kronnerwetter C , et al. Long-term evolution of multiple sclerosis iron rim lesions in 7T MRI. Brain 2021; 144(3): 833–847. 33484118 10.1093/brain/awaa436

[16] Polman CH Reingold SC Banwell B , et al. Diagnostic criteria for multiple sclerosis: 2010 revisions to the McDonald criteria. Ann Neurol 2011; 69(2): 292–302. 21387374 10.1002/ana.22366 PMC3084507

[17] Polman CH Reingold SC Edan G , et al. Diagnostic criteria for multiple sclerosis: 2005 revisions to the ‘McDonald criteria’. Ann Neurol 2005; 58(6): 840–846. 16283615 10.1002/ana.20703

[18] Kurtzke JF . Rating neurologic impairment in multiple sclerosis. Neurology 1983; 33(11): 1444–1452. 6685237 10.1212/wnl.33.11.1444

[19] Manouchehrinia A Westerlind H Kingwell E , et al. Age-related multiple sclerosis severity score: Disability ranked by age. Mult Scler 2017; 23(14): 1938–1946. 28155580 10.1177/1352458517690618 PMC5700773

[20] Hurley AC Al-Radaideh A Bai L , et al. Tailored RF pulse for magnetization inversion at ultrahigh field. Magn Reson Med 2010; 63(1): 51–58. 19859955 10.1002/mrm.22167

[21] Renard D Castelnovo G Bousquet PJ , et al. Brain MRI findings in long-standing and disabling multiple sclerosis in 84 patients. Clin Neurol Neurosurg 2010; 112(4): 286–290. 20061078 10.1016/j.clineuro.2009.12.012

[22] Giorgio A Stromillo ML Bartolozzi ML , et al. Relevance of hypointense brain MRI lesions for long-term worsening of clinical disability in relapsing multiple sclerosis. Mult Scler 2014; 20(2): 214–219. 23877971 10.1177/1352458513494490

[23] Mistry N Tallantyre EC Dixon JE , et al. Focal multiple sclerosis lesions abound in normal appearing white matter. Mult Scler 2011; 17(11): 1313–1323. 21788249 10.1177/1352458511415305

[24] Elliott C Belachew S Wolinsky JS , et al. Chronic white matter lesion activity predicts clinical progression in primary progressive multiple sclerosis. Neurology 2012; 142(4): 2787–2799. 10.1093/brain/awz212 PMC6736181 31497864

[25] Yao B Bagnato F Matsuura E , et al. Chronic multiple sclerosis lesions: Characterization with high-field-strength MR imaging. Radiology 2012; 262(1): 206–215. 22084205 10.1148/radiol.11110601 PMC3244667

[26] Yushkevich PA Piven J Hazlett HC , et al. User-guided 3D active contour segmentation of anatomical structures: Significantly improved efficiency and reliability. Neuroimage 2006; 31(3): 1116–1128. 16545965 10.1016/j.neuroimage.2006.01.015

[27] Mackinnon DP Fairchild AJ Fritz MS . Mediation analysis. Annu Rev Psychol 2007; 58: 593. 16968208 10.1146/annurev.psych.58.110405.085542 PMC2819368

[28] Weber CE Krämer J Wittayer M , et al. Association of iron rim lesions with brain and cervical cord volume in relapsing multiple sclerosis. Eur Radiol 2022; 32(3): 2012–2022. 34549326 10.1007/s00330-021-08233-w PMC8831268

[29] Zhang S Nguyen TD Hurtado Rúa SM , et al. Quantitative susceptibility mapping of time-dependent susceptibility changes in multiple sclerosis lesions. AJNR Am J Neuroradiol 2019; 40(6): 987–993. 31097429 10.3174/ajnr.A6071 PMC6565472

[30] Mehta V Pei W Yang G , et al. Iron is a sensitive biomarker for inflammation in multiple sclerosis lesions. PLoS ONE 2013; 8(3): e57573. 10.1371/journal.pone.0057573 PMC3597727 23516409

[31] Blindenbacher N Brunner E Asseyer S , et al. Evaluation of the ‘ring sign’ and the ‘core sign’ as a magnetic resonance imaging marker of disease activity and progression in clinically isolated syndrome and early multiple sclerosis. Mult Scler J: Exp Transl Clin 2020; 6(1): 15480. 10.1177/2055217320915480 PMC7132556 32284875

[32] Clarke MA Pareto D Pessini-Ferreira L , et al. Value of 3T susceptibility-weighted imaging in the diagnosis of multiple sclerosis. AJNR: Am J Neuroradiol 2020; 41(6): 1001–1008. 32439639 10.3174/ajnr.A6547 PMC7342768

